# Comparative Transcriptome-Based Mining of Senescence-Related MADS, NAC, and WRKY Transcription Factors in the Rapid-Senescence Line DLS-91 of *Brassica rapa*

**DOI:** 10.3390/ijms22116017

**Published:** 2021-06-02

**Authors:** So Young Yi, Jana Jeevan Rameneni, Myungjin Lee, Seul Gi Song, Yuri Choi, Lu Lu, Hyeokgeun Lee, Yong Pyo Lim

**Affiliations:** 1 Institute of Agricultural Science, Chungnam National University, Daejeon 34134, Korea; yisy@cnu.ac.kr (S.Y.Y.); saijeevan7@gmail.com (J.J.R.); anova03@hanmail.net (M.L.); 2 Molecular Genetics and Genomics Laboratory, Department of Horticulture, College of Agriculture and Life Science, Chungnam National University, Daejeon 34134, Korea; ssong5253@gmail.com (S.G.S.); choiyuri33@kaist.ac.kr (Y.C.); dlLL220186@126.com (L.L.); gurrmsdl@nongwoobio.co.kr (H.L.)

**Keywords:** *Brassica rapa*, DLS, DLS-91, comparative transcriptome, AGL-MADS, NAC, WRKY, transcription factors

## Abstract

Leaf senescence is a developmental process induced by various molecular and environmental stimuli that may affect crop yield. The dark-induced leaf senescence-91 (DLS-91) plants displayed rapid leaf senescence, dramatically decreased chlorophyll contents, low photochemical efficiencies, and upregulation of the senescence-associated marker gene *BrSAG12-1*. To understand DLS molecular mechanism, we examined transcriptomic changes in DLS-91 and control line DLS-42 following 0, 1, and 4 days of dark treatment (DDT) stages. We identified 501, 446, and 456 DEGs, of which 16.7%, 17.2%, and 14.4% encoded TFs, in samples from the three stages. qRT-PCR validation of 16 genes, namely, 7 MADS, 6 NAC, and 3 WRKY, suggested that *BrAGL8-1*, *BrAGL15-1*, and *BrWRKY70-1* contribute to the rapid leaf senescence of DLS-91 before (0 DDT) and after (1 and 4 DDT) dark treatment, whereas *BrNAC046-2*, *BrNAC029-2/BrNAP*, and *BrNAC092-1/ORE1* TFs may regulate this process at a later stage (4 DDT). In-silico analysis of cis-acting regulatory elements of *BrAGL8-1, BrAGL42-1, BrNAC029-2, BrNAC092-1,* and *BrWRKY70-3* of *B. rapa* provides insight into the regulation of these genes. Our study has uncovered several AGL-MADS, WRKY, and NAC TFs potentially worthy of further study to understand the underlying mechanism of rapid DLS in DLS-91.

## 1. Introduction

Leaf senescence is a complex, programmed series of events involving various physiological and molecular processes, including chlorophyll degradation and a decline in photochemical efficiency [1,2]. Senescence in plants, which occurs through nutrient mobilization and recycling [3,4,5], is induced by biotic factors, such as aging and pathogen attack, and by abiotic factors, including extreme temperatures, nutrient deficiency, insufficient light, darkness, and drought [6].

Chinese cabbage (*Brassica rapa* ssp. *pekinensis*) is a leafy vegetable that generally grows best from late summer to early winter but that can also be cultivated year-round depending on the genotype. Chinese cabbage is readily affected by various external environmental factors that lead to multiple nutrient losses, cell death-induced leaf yellowing, weight loss due to reduced relative humidity, and decomposition [7,8]. In Chinese cabbage, the leaf is the economically most important part of the plant; consequently, leaf senescence is responsible for major crop yield losses. Nevertheless, studies on leaf senescence in Chinese cabbage are limited or lacking [9].

To study senescence, dark conditions are often used because they induce rapid, simultaneous senescence in detached leaves, similar to that observed during normal plant development [10,11]. In addition, many functional senescence associated genes (SAGs) are involved in some of the same mechanisms during both age-triggered and dark-induced senescence [12].

In senescing leaves, leaf chlorosis is a visible symptom of programmed cell death and other degenerative metabolisms [13,14]. The chlorophyll (Chl) degradation process occurs through a multiphase pathway. Stay-green 1 (SGR1), Chl catabolic enzymes (CCEs), and light-harvesting complex II (LHCII) form a protein interaction complex for chlorophyll degradation during senescence. This complex removes magnesium and phytol from the chlorophyll degradation process via magnesium-dechelatase and chlorophyllase, respectively. Pheophorbide is converted to pFCC-1, which is subsequently transformed into non-fluorescent Chl catabolites (NCCs). The chlorophyll degradation process therefore produces colorless NCCs and changes the color of leaves [15,16,17,18]. Several catabolic genes, namely, chlorophyll b reductase (CBR) [19], chlorophyllase (CLH) [20], pheophorbide a oxygenase (PaO) [21], RCC reductase (RCCR) [22], pheophorbidase (PPD) [23], and stay-green (SGR/SID) [13,24], are involved in Chl degradation.

NGS technologies, such as transcriptome sequencing and microarray-based analysis of global gene expression, have been successfully applied for understanding the molecular mechanisms of leaf senescence in different crop species. For example, Lu et al. [25] identified 881 transcription factors (TFs), mainly belonging to bHLH, MYB, ERF, MYB-related, NAC, and WRKY families, in the seasonal leaf-senescence transcriptome of black cottonwood. In sunflower, a comparative transcriptome study uncovered genotype-specific genes and TFs related to leaf senescence [26]. In monocot plants such as maize, members of CO-like, NAC, ERF, GRAS, WRKY, and ZF-HD TF families have been found to be involved in early leaf senescence [27].

Multiple studies have described the functions of leaf senescence-associated TFs, which mainly include NACs and WRKYs along with a few MADS family members. For example, *ORE1/ANAC092* plays crucial roles in aging leaf and flower tissues [28,29], and the *NAP2* gene positively regulates leaf senescence by directly binding to SAGs in tomato plants [30]. In addition, Oda-Yamamizo and colleagues have shown that ANAC046 promotes leaf senescence in Arabidopsis by activating Chl catabolic genes and SAGs [31]. Other studies have been conducted on the role of NAC TFs in the regulation of leaf senescence [4,32].

In addition, to the above-mentioned genes, the WRKY gene *CpWRKY71* (*Chimonanthus praecox* *WRKY71*) has been found to positively regulate senescence and flowering in Arabidopsis [33]. Another study on *AtWRKY42* revealed that this gene promotes leaf senescence by modulating the transcription of SAGs and genes involved in SA and ROS synthesis [34]. Analysis of mutant Arabidopsis lines of *WRKY45* has demonstrated that this gene binds to RGA-LIKE1 (RGL1) and promotes age-triggered leaf senescence [35]. Various other studies have uncovered the roles of WRKY genes as central regulators during leaf senescence in cotton (*GhWRKY17* [36], *GhWRKY27* [37], *GhWRKY42* [38], and *GhWRKY91* [39]), wheat (*TaWRKY42-B* [40]), Arabidopsis (*AtWRKY55* [41] and *AtWRKY75* [42]), and Chinese flowering cabbage (*BrWRKY65* [43]).

At present, only a few MADS-box TFs, which are primarily known as flowering developmental regulators [44], have been functionally associated with leaf senescence. A few studies have identified MADS-box genes with functions related to leaf senescence. For instance, overexpression of the FYFL MADS-box gene delays leaf and sepal senescence in tomato [45]. A global gene expression analysis during different leaf senescent stages revealed the differential expressions of *AtAGL8 (AT5G60910)* and *AtAGL20 (AT2G45660)* [46]. Likewise, Fang et al. [47] have reported that overexpression of *AGL15* delays *SAG12* promoter activity and slows the senescence of sepal, petal, and silique tissues in Arabidopsis. Furthermore, TFs characterized as having a role in various trans-regulation, signaling, metabolic, and hormonal responses of senescence can be found in the LSD leaf senescence database (https://bigd.big.ac.cn/lsd/index.php, accessed on 17 March 2021) [48].

Leaf senescence is a complex process arising from cell death in leaf tissues. In our initial study reported here, we adopted different experimental approaches to identify factors causing leaf yellowing in Chinese cabbage.

We selected two genotypes exhibiting different senescence during dark-treatment senescence: DLS-42 (used as a control) and the rapid-senescence line DLS-91. To identify the cause of early senescence in DLS-91, we mainly focused on physiological factors, such as chlorophyll content and photochemical efficiency, as well as possible genetic factors identified by RNA-seq analysis.

## 2. Results

### 2.1. DLS-91 Exhibited More Rapid Leaf Senescence than the Control Line during Dark Treatment

**Figure 1:**
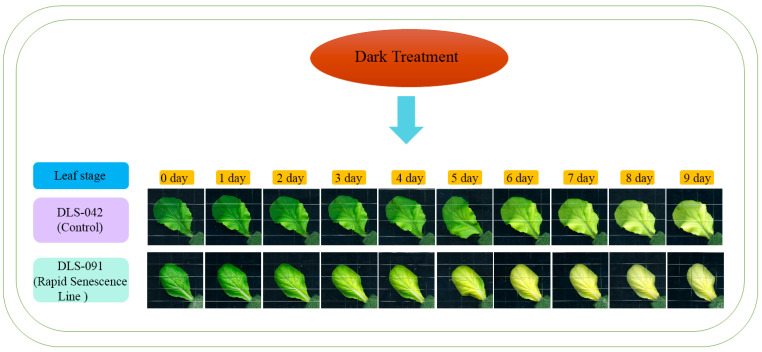
Dark induced leaf senescence in DLS-42 and DLS-91. Leaf senescence is divided into three phases: I (days 0–3), II (days 4–6), and III (days 7–9).

**Figure 2:**
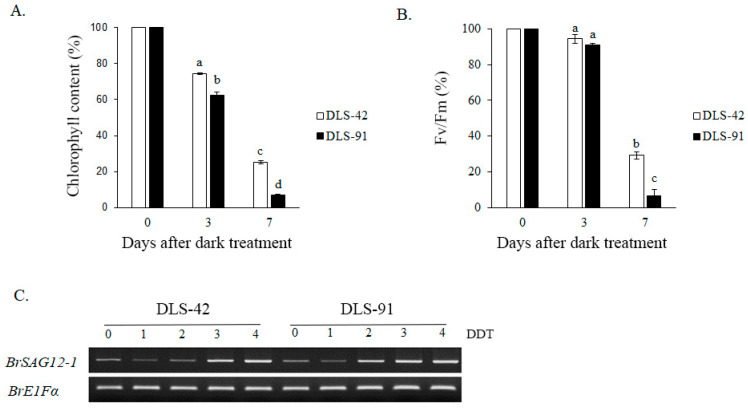
Phenotypic changes during dark-induced leaf senescence. Variation in (**A**) chlorophyll content, (**B**) photochemical efficiency *(*F_v_/F_m_), and (**C**) expression of the senescence-related marker SAG12-1 (with EF1-α as a reference gene). Data is the mean of three independent experiments. Error bars represent standard deviation of three biological replicates. Different letters indicate significant differences. One-way ANOVA test is performed using SPSS software. DAT, days of dark treatment.

Because yellowing is the most distinct visual phenotype associated with leaf senescence, we used leaf color to compare different stages of the dark-induced leaf senescence process. To identify phenotypic changes occurring in *B. rapa* under dark treatment, we selected two inbred lines, the control line DLS-42 and the rapid-senescence line DLS-91, according to criteria specified in Materials and Methods. Similar to the protocol used in an earlier study in Arabidopsis [49], fourth leaves were detached from the two genotypes and incubated in darkness for up to 9 days (Figure 1). Leaf yellowing was first observed on the third day of dark treatment (phase I) in DLS-91 and on the sixth day in DLS-42 (Figure 1). By day 9, leaves of DLS-91 were entirely yellow; in contrast, the leaf color of DLS-42 on the final day was similar to that of DLS-91 on 5-DDT (Figure 1). Therefore, to further detect leaf senescence symptoms, we selected 0-, 3-, and 7-DDT samples based on leaf color and analyzed chlorophyll content and photochemical efficiency (Fv/Fm) (Figure 1). After 3 DDT, the reduction in the chlorophyll content of DLS-91 (ca. 40%) was higher than that of DLS-42 (ca. 25%); by day 7, chlorophyll was totally depleted from DLS-91 (ca. 97%) compared with the control (ca. 71%) (Figure 2B). Similarly, phytochemical efficiency began to drop at 3 DDT and was completely reduced in DLS-91 leaves compared with those of DLS-42 at 7 DDT (Figure 2C). According to these phenotypical and physiological parameters, leaf senescence occurs earlier in DLS-91 than in DLS-42 (Figure 1 and Figure 2A,B). In addition, analysis of the expressions of six senescence-related genes [32,50], namely, *Ethylene Insensitive 2* (*BrEIN2*), *Senescence-associated gene 12* (*BrSAG12-1* and *BrSAG12-2*), and *ORESARA 1* genes *BrORE1-1* and *BrORE1-2*, revealed that *BrSAG12-1* was highly differentially expressed at 2 DDT between DLS-91 and DLS-42 (Figure 2C and Figure S3). Taken together, all of these results indicate that DLS-91 undergoes earlier senescence during dark treatment compared with DLS-42.

### 2.2. Assessment of DLS-42 and DLS-91 RNA Sequence Data and Identification of Stage- and Genotype-Specific Genes

In this study, the first to address dark-induced leaf senescence at the transcriptomic level in *B. rapa*, we identified the complete set of differentially expressed genes (DEGs) during dark treatment in DLS-91. Previous transcriptomic analyses of leaf senescence in Arabidopsis used 0–3-DDT leaf tissues and were able to identify important genes involved in leaf senescence [49,51]. In contrast, we carried out RNA sequencing, with three biological replicates, of 0-, 1-, and 4-DDT leaves of DLS-42 and DLS-91 (Figure 1), which allowed genes involved in early and late stages of DLS to be identified in the rapid-senescence line. On average, 18 to 20 million raw reads were generated from both genotypes (DLS-42 and DLS-91). Q20 and Q30 percentages across all reads were more than 97% and 94%, respectively, with a sequencing error rate of 0.06%, and the average mapping rate for all samples of DLS-42 and DLS-91 lines was between 87% and 89%. Other important aspects of the sequencing data are statistically summarized in Table S1.

**Figure 3:**
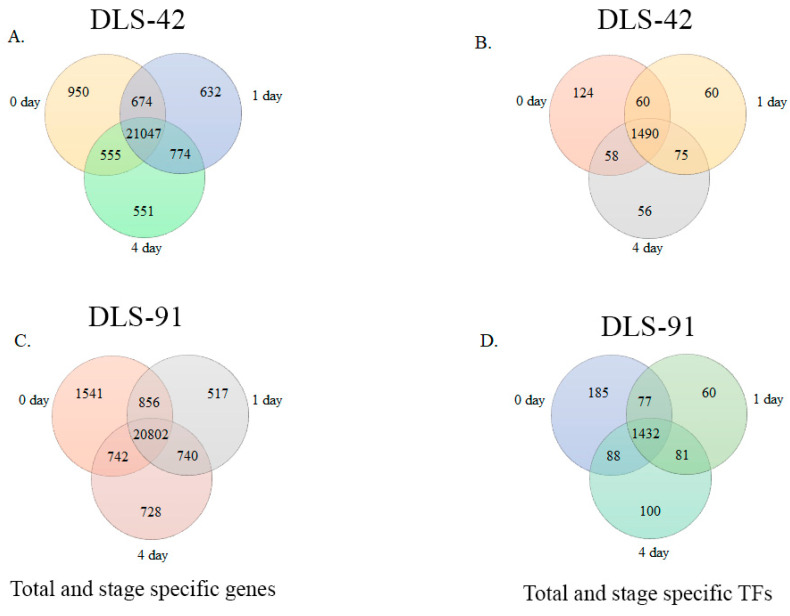
Venn diagram analysis of DLS-42 and DLS-91 transcriptome data. (**A**,**C**) Venn diagrams of total and stage-specific genes. (**B**,**D**) Venn diagrams of total and stage-specific transcription factors predicted in DLS-42 and DLS-91 leaf tissues.

We identified 21,047 genes in DLS-42 that had a fragments per kilobase of transcripts per million mapped reads (FPKM) value > 1; among them, 4.5%, 3%, and 2.6% were expressed in 0-, 1-, and 4-DDT leaf samples, respectively (Figure 3A and Table S2). These genes included 1490 TF genes, of which 8.3%, 4%, and 3.7% were specifically expressed in 0-, 1-, and 4-DDT leaf samples (Figure 3B). In the rapid-senescence line DLS-91, we identified 20,802 genes with FPKM values > 1, among which 7.4%, 2.5%, and 3.4% were significantly expressed in 0-, 1-, and 4-DDT leaves (Figure 3C). In addition, we identified 1432 TFs, of which 12.9%, 4.2%, and 7% were detected in 0-, 1-, and 4-DDT leaf samples (Figure 3D and Table S3). Other significantly expressed genes, including functionally uncharacterized, genotype-specific, and newly annotated (i.e., without a v1.5 *B. rapa* ID) genes and TFs found in the two genotypes at different stages, are summarized in Tables S2 and S3.

### 2.3. Identification of DEGs and TF Genes in 0-, 1-, and 4-Day Dark-Treated Samples

**Figure 4:**
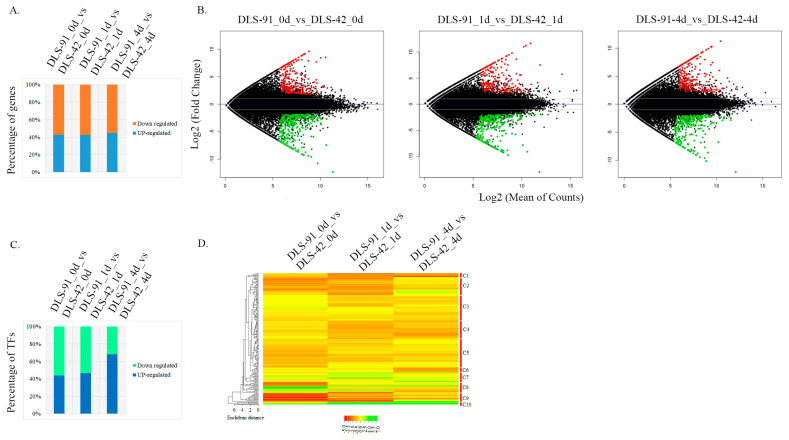
Identification of differentially expressed genes and transcription factors from the transcriptomes of DLS-42 and DLS-91 (0, 1, and 4 days of dark treatment). (**A**) Bar chart showing relative proportions of up- and downregulated genes. (**B**) MA plot of differentially expressed genes. The x-axis indicates log_2_ (mean number of counts), and the y-axis corresponds to log_2_ fold change. (**C**) Bar chart showing relative proportions of up- and downregulated transcription factors. (**D**) Heat map of differentially expressed transcription factors identified from the transcriptome data. The transcription factors are arranged according to their positions in the cladogram.

Identification of DEGs and differentially expressed TFs (DETFs) from the entire set of transcriptome data was performed by comparing the two genotypes at each treatment stage, i.e., DLS-91_0 vs. DLS-42_0, DLS-91_ 1 vs. DLS-42_1, and DLS-91_4 vs. DLS-42_4. The DEGs and DETFs, which were retrieved according to the criteria of false discovery rate (FDR) < 0.01 and log_2_ fold change (FC) > 1, were classified by stage and are summarized in Tables S4 and S5. Among the 501, 446, and 456 DEGs identified in leaves after 0, 1, and 4 DDT, respectively, 42% to 44% were upregulated, and 55% to 57% were downregulated in DLS-91 relative to DLS-42, with the highest number of upregulated genes detected in 4-DDT leaves (Figure 4A). This differentiation in gene expression patterns can be clearly seen in the MA plot in Figure 4B, which depicts genes with log_2_ FC ≥ 1 in red and those with log_2_ FC < 0 in green. Furthermore, 89 DEGs in 4-DDT leaves and 80 DEGs each in 0- and 1-DDT leaves had log_2_ FC values > 5; these genes were annotated as having various molecular functions related to plant growth and development (Table S4). A total of 212, 136, and 139 genes in 0-, 1-, and 4-DDT samples, respectively, were specifically differentially expressed between DLS-91 and DLS-42; similarly, 236, 245, and 266 genes were specifically differentially expressed in 0 vs. 1 DDT, 0 vs. 4 DDT, and 1 vs. 4 DDT samples, respectively (Table S4). In the 0-, 1-, and 4-DDT samples, respectively, 71, 61, and 58 genes were novel and not yet functionally characterized, whereas 42, 36, and 37 were specific to *B. rapa* (i.e., lacking an *Arabidopsis thaliana* ID). Furthermore, 18, 16, and 16 genes were newly annotated in the *B. rapa* genome (i.e., lacking a v1.5 *B. rapa* ID); most of these had a log_2_ FC > 3 in samples at all three stages (0, 1, and 4 DDT) (Table S4).

A total of 33 TF families were identified in the transcriptome. The largest number of TFs, 29, belonged to the NAC family, followed by 24 each in MYB and WRKY, 13 in MADS, 9 each in bHLH and ERF, 6 each in ARR-B, HB-other, and HD-ZIP, and 5 each in ARF, C2H2, C3H, CO-Like, G2-Like, and Trihelix families. Each of the remaining TF families were represented by fewer than five TF genes. In total, 84, 77, and 66 DETFs were detected at 0-, 1-, and 4-DDT leaf stages, respectively (Table S5). The highest percentage of upregulated TFs (65%) was detected in 4-DDT samples, which was followed by 47.6% and 38.3% in 0- and 1-DDT leaf samples, respectively; in contrast, the highest percentage of downregulated TFs was found in stage-1 samples (Figure 4C). Using the CIMminer online tool, we constructed a gene expression heat map based on the DETF FC values and classified the genes into 10 clusters (C1–C10) (Figure 4D). Among these clusters, TFs in cluster C10 were relatively highly expressed in DLS-91 at all three stages, whereas most TF genes in clusters C7 and C8 had their highest expressions in DLS-91 during stages 1 (DLS-91_ 1 vs. DLS-42_1) and 0 (DLS-91_0 vs. DLS-42_0), respectively (Figure 4D). Relative expressions of the other TFs varied at different time points in DLS-91. Some, such as *WRKY38*, *BrAGL15-1*, and *BrAGL8-1*, displayed steady upregulation, whereas a few were upregulated only at two stages, namely, *MYB116* and *RSM3* at 0 and 1 DDT, and *SAP12, WRKY50*, and *WRKY 62* at 1 and 4 DDT. The remaining upregulated genes exhibited stage-specific expressions: *MYB29*, *MEE3*, *BMY5*, *ARF18*, *RSM1*, and *BraA06g029890* at 0 DDT; *MYB16*, *BraA04g030300*, and *BraA02g007650* at 1 DDT; and *BraA08g003770*, *SHP1*, *BraA04g002050*, and *BraA08g019170* at 4 DDT (Table S5). Additional information, including expression (FC) data and gene and TF annotations, is provided in Tables S4 and S5. Genes newly identified in this analysis and those exhibiting genotype- and stage-specific expressions should be a useful resource for spatiotemporal functional characterization related to DLS in *B. rapa* DLS-91.

### 2.4. Functional Enrichment Analysis of DEGs

**Figure 5:**
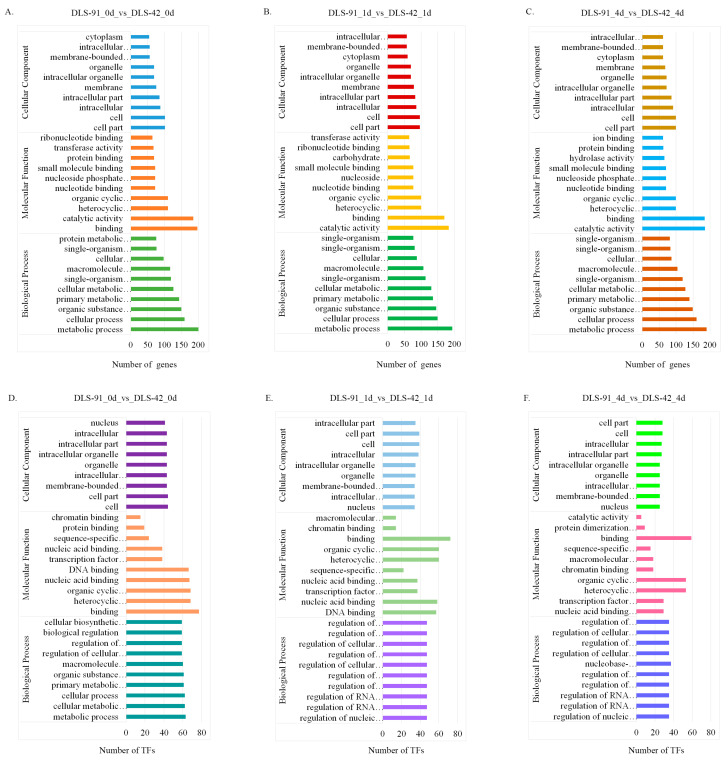
Gene ontology classification of differentially expressed genes (DEGs) and transcription factors (TFs) identified in DLS-42 and DLS-91 leaf samples subjected to 0, 1, and 4 days of dark treatment. (**A**–**C**) DEGs. (**D**–**F**) TFs. The number of DEGs and TFs assigned to different GO terms in biological process, cellular component, and molecular function categories using the agriGO online database are shown.

The genes and TFs found to be differentially expressed at each treatment stage were subjected to gene ontology (GO) annotation using the agriGO database (http://systemsbiology.cau.edu.cn/agriGOv2/, accessed on 17 March 2021). The top 10 GO terms from each of three categories (biological process, molecular function, and cellular component) were used to generate the graphs shown in Figure 5. GO terms were assigned to 362, 419, and 413 DEGs from 0-, 1-, and 4-DDT samples, respectively, and are listed in Table S6. In the biological process category, most DEGs were related to “metabolic process”, followed by “cellular process” and “organic substance metabolic process”. Similarly, most genes related to molecular function were involved in “catalytic activity”, followed by “binding” and “organic and heterocyclic compound binding”. Under the cellular component category, many DEGs were localized to “cell”, “cell part”, and “intracellular” components (Figure 5A–C). TFs at the three leaf senescence stages were associated with the 84, 77, and 72 GO terms listed in Table S7. Under the biological process category, most TFs detected at 0 DDT were assigned to “metabolic process”, followed by “cellular and cellular metabolic process”. At 1 and 4 DDT, in contrast, TFs were enriched in 10 biological process terms, such as “regulation of biosynthetic process and regulation of cellular biosynthetic process”. In regard to molecular function, most TFs were enriched in “binding” and “organic and heterocyclic compound binding” at all sampled stages. The most heavily enriched cellular component terms were “cell” “cell part” and “organelle” (Figure 5D–F).

To identify pathways associated with genes upregulated and downregulated at each leaf senescence stage, we performed a KEGG pathway enrichment analysis on the entire set of DEGs (Table S8). As a result, genes upregulated at 0, 1, and 4 DDT were assigned to 67, 48, and 59 pathways, respectively. Most of these genes were involved in metabolic and secondary metabolite biosynthetic pathways, followed by glycolysis/gluconeogenesis and DNA replication-related pathways. In addition, a few genes differentially expressed at 0 and 1 DDT were related to terpenoid and chlorophyll biosynthesis. Similarly, several DEGs at 1 and 4 DDT were involved in fatty acid biosynthesis and degradation, while others up- or downregulated at 0 and 4 DDT were associated with amino acid degradation (Table S8). In addition, genes downregulated at 0, 1, or 4 DDT were found to participate in 61, 65, and 65 pathways, respectively, whereas genes expressed continuously at all three sampled stages were involved in pathways related to metabolism, secondary metabolite biosynthesis, and purine, carbon, and propanoate metabolisms (Table S8).

### 2.5. Transcript Abundance of Genes with Various Functions during Senescence

In general, leaf senescence comprises a series of molecular reactions involving various genes and TFs at different stages [52]. To excavate the entire set of senescence-associated genes (SAGs) in DLS-91, we assembled a comprehensive set of genes and TFs with functions related to leaf senescence in different plant species [1,4,48,51,53,54,55,56,57,58,59], including leaf coloration, chlorophyll biosynthesis and degradation, photosystems I and II, phytohormone signal components, chromatin mediated regulation, transcription and post-transcription, translation and post-translation mediated regulators, leaf senescence and plant immunity, and genes that are upregulated and downregulated during DLS. In total, 3003 orthologous genes with a wide range of expression levels among different time frames were identified from the complete transcriptome data (Table 1 and Table S9).

**Figure 6:**
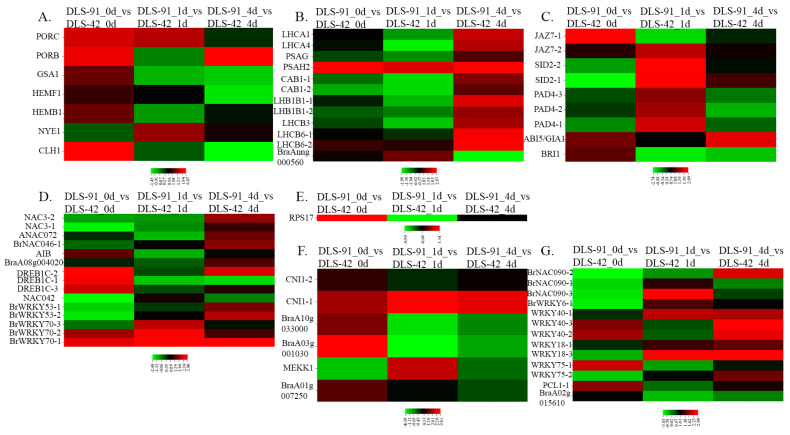
Expression heat maps of genes associated with various functions at different stages of dark-induced leaf senescence (0, 1, and 4 days of dark treatment). Genes associated with (**A**) chlorophyll biosynthesis and degradation, (**B**) photosystems I and II, (**C**) phytohormones signal components, (**D**) transcriptional regulators, (**E**) regulators of translation, (**F**) post-translational regulators, and (**G**) leaf senescence and plant immunity are shown.

Out of 55 chlorophyll-related genes identified from the transcriptome data, 44 were related to chlorophyll biosynthesis, and 11 were chlorophyll-degrading genes. Five of the chlorophyll biosynthetic genes, *PORB*, *PORC*, *GSA1*, *HEMB1*, and *HEMF1*, were highly expressed (log_2_ FC > 1) at 0 DDT in DLS-91 compared with DLS-42, with *PORC* and *PORB* also highly expressed at 1 DDT and 4 DDT, respectively (Figure 6A). Among chlorophyll-degrading genes, *CLH1* was highly expressed at 0 DDT in DLS-91 compared with the remaining two stages and with DLS-42. In contrast, *NYE1* was more strongly expressed during later stages (1 and 4 DDT) in DLS-91 (Figure 6A). In addition, expression levels of chlorophyll-degrading genes and the rate of chlorophyll content reduction in leaves were higher in DLS-91 than in DLS-42. Overall, these results indicate that chlorophyll biosynthetic and degradation functions were activated earlier in DLS-91 than in DLS-42 (Figure 2A; Table S9).

A total of 55 genes related to photosystems I and II were also identified from the transcriptome data. Among them, 11 genes—*PSAH2*, *LHCA1*, *LHCA4*, and *PSAG* of PSI and two *LHCB6*, two *LHB1B1*, two *CAB1* and one *LHCB3* gene(s) of PSII—were highly expressed (log_2_ FC > 1) at the late stage (4 DDT) in DLS-91 (Figure 6B). In addition, the *PSAH2* gene of PSI was highly expressed (log_2_ FC > 1) at all treatment stages (0–4 DDT), and *BraAnng000560* of PSII exhibited stage-specific expression (at 1 DDT) in DLS-91 compared with DLS-42 (Figure 6B).

We also identified 29 genes related to phytohormones, auxin, abscisic acid, brassinosteroids, cytokinins, ethylene, abscisic acid, jasmonic acid, and salicylic acid. In regard to the three sampled stages, the largest number of genes (*JAZ7-2*, 2 *SID2, SID2-2*, 3 *PAD4*, *PAD4-2,* and *PAD4-3*) were more highly expressed in 1-DDT samples in DLS-91 compared with DLS-42 (Figure 6C), with *JAZ7-1* and *BRI1* more strongly expressed at 0 DDT as well (Table S9). This result indicates that hormones such as jasmonic acid, abscisic acid, and brassinosteroids are produced early in DLS-91—at 0 DDT, whereas salicylic acid is produced during dark treatment (1 DDT) in this genotype. Consistent with these findings, Yin et al. [60] have suggested that SA delays leaf senescence by regulating concentrations of methyl jasmonates. In Arabidopsis, *ABI5* has been determined to delay DLS [61]. In our study, the expression FC of the ortholog of *ABI5/GIA1* (*BraA05g009140*) was higher at all sampled treatment stages in DLS-91.

Genes shown to regulate senescence at the RNA/protein level (transcription, translation, and post-translation) in a previous investigation [56] were highly expressed in DLS-91 during at least two sampled stages in our study (Figure 6D–F). In addition, a large number of genes related to transcriptional regulation during leaf senescence were highly expressed at 4 DDT in DLS-91 samples, which indicates that these genes might be involved, even in this genotype, in regulating senescence by controlling the transcription of their targets [56].

In addition to the above findings, the TFs shown in Figure 6G have been reported to play a role in leaf senescence and plant immunity [54]. Most of these orthologous genes showed enhanced expression after dark treatment, thus indicating that these genes were activated at early stages of DLS in DLS-91.

Totally 194 genes are downloaded from the LSD leaf senescence database included genes related to senescence and leaf color, most of which had their maximum expressions at 0 DDT (Table S9). Using age-triggered and dark-induced leaf senescence genes previously identified from the Arabidopsis transcriptome [1], we identified 2528 orthologs from our transcriptome data. Similar to previous expression data, the genes in our study exhibited a broad range of expression levels at different stages and between DLS-42 and DLS-91. In addition, these genes were up- or downregulated in a genotype-dependent manner (Table S9).

### 2.6. Identification of TFs Involved in Senescence

We additionally identified 182 TFs with a broad spectrum of functions related to senescence (Table S9). Among them, 67 genes have been reported to participate in senescence on the basis of published mutant or transgene expression data [10,28,29,31,35,46,47,62,63,64,65,66,67,68,69,70,71,72,73,74,75,76,77,78,79] (Table S10). Ten of 11 TFs of the bHLH family, which are reported to regulate genes at the transcription level during senescence [56], did not shown any significant expression variation at any stages or between genotypes; the exception was *BrAIB1-2*, which was more highly expressed at 0 DDT in DLS-91 than in DLS-42. Of the two identified bZIP genes, *BrABI5-2* was more strongly expressed at 1 and 4 DDT in DLS-91 compared with DLS-42 (Table S10). Interestingly, 10 *AGAMOUS-LIKE* (*AGL*)-MADS-box genes *(BrAGL8-1, BrAGL8-2*, *BrAGL8-3, BrAGL15-1, BrAGL15-2, BrAGL20-1*, *BrAGL20-2, BrAGL20-3, BrAGL42-1,* and *BrAGL42-2)*, had diverse expression patterns between DLS-42 and DLS-91 at all stages. According to the LSD database, eight of these genes participate in leaf color regulation, and *AGL15* is involved in the regulation of leaf senescence in Arabidopsis (Table S10) [48,57]. Two of these genes, *BrAGL8-1*, and *BrAGL15-1*, were more strongly expressed in DLS-91 than in DLS-42 at all stages, whereas *BrAGL20-1* was more highly expressed in DLS-91 only at 1 and 4 DDT. In addition, *BrAGL8-3*, the two *BrAGL42* genes, and two of the *BrAGL20* genes (*BrAGL20-2* and *BrAGL20-3*) were more strongly expressed in DLS-42 than in DLS-91 at all stages (Table S10). Although the 21 identified NAC genes exhibited a wide range of expressions, most did not have genotype-specific expressions. In addition, log_2_ FC values of *BrNAC03-1*, *BrNAC046-1*, *BrNAC083-2*, *BrNAC090-2,* and *BrNAC090-3* ranged between 1 and 3 at both dark-treatment stages (1 and 4 DDT) in DLS91 (Table S10). Furthermore, two ERF family *BrDREB2A* genes, *BrDREB2A-1* and *BrDREB2A-3*, were highly expressed at 0 and 4 DDT in DLS-91. Consistent with our results, these two genes have been reported to be upregulated in response to dark-induced leaf senescence in Arabidopsis [1]. Five out of 11 WRKY genes identified in our study were highly expressed in either of the stages of DLS-91. The relative expressions of the six remaining WRKY genes (3 each of *BrWRKY54* and *BrWRKY70*) specifically highly expressed in DLS-91 (Table S10). Finally, the expression of the only identified zinc-finger family TF gene, *PKDM7D*, which is reported to function in leaf senescence and plant immunity, did not vary in either DLS-42 or DLS-91 at any stage (Table S10).

### 2.7. qRT-PCR Validation of MADS, NAC, and WRKY TFs

**Figure 7:**
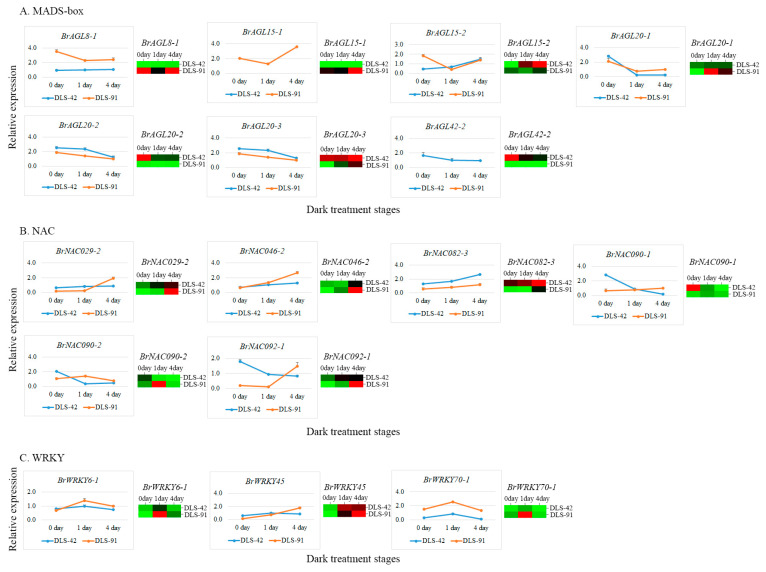
Expression validation of transcription factors associated with senescence in dark-treated leaf samples collected after 0, 1, and 4 days of treatment. (**A**) MADS-box, (**B**) NAV, and (**C**) WRKY expressions. The line graph and the heat map indicate qRT-PCR- and RNA sequencing-based expression levels, respectively.

To further validate gene transcript abundances, we selected 16 genes (seven *AGL*, six *NAC*, and three *WRKY* genes) (Table S11) on the basis of their expression levels (FPKM values) and roles in leaf color change and leaf senescence (Table 2). The transcript abundance of *BrAGL8-1* was at least seven times higher at 0, 1, and 4 DDT in DLS-91 compared with DLS-42, and a similar expression pattern was observed in the qRT-PCR analysis (Figure 7A). Two paralogs of *BrAGL15 (BrAGL15-1* and *BrAGL15-2)* had contrasting expression patterns based on the RNA-seq data. Likewise, the qRT-PCR analysis indicated that the expression of *BrAGL15-1* was genotype specific (i.e., limited to DLS-91) at all stages, whereas *BrAGL15-2* was slightly more highly expressed in DLS-91 at 0 DDT than at the other two sampled stages (Figure 7A and Table 2). Similarly, *BrAGL20-2* and *BrAGL20-3* paralogs of *BrAGL20* exhibited genotype (DLS-42)-specific expression at all stages according to the transcriptome data, whereas *BrAGL20-1* was slightly more highly expressed at 0 DDT in DLS-42 and at 1 and 4 DDT in DLS-91. The overall results of the qRT-PCR analysis of *BrAGL20* genes were well correlated with the transcriptome expression data (Figure 7A and Table 2). Finally, *BrAGL42-2* exhibited higher transcript abundance in DLS-42 than in DLS-91 at all stages, and qRT-PCR also revealed its DLS-42-specific expression (Figure 7A and Table 2). The expression patterns of *AGL* genes uncovered in this study suggest that these genes have completely genotype (DLS-42/DLS-91)-specific functions during DLS.

Among the analyzed NAC genes, *BrNAC029-2* was approximately five times more highly expressed in 0- and 1-DDT samples of DLS-42 than in DLS-91 but was more strongly expressed in 4-DDT samples of DLS-91 than those of DLS-41. The results of qRT-PCR validation of this gene were correlated with the transcriptome data (Figure 7B and Table 2). The *BrNAC046-2* gene was highly expressed at 1 and 4 DDT in DLS-91, and qRT-PCR-based relative expression levels of this gene were consistent with its transcript abundance (Figure 7B and Table 2). Although transcripts of *BrNAC082-3* accumulated at all stages in both genotypes, this gene was more highly expressed in DLS-42 than in DLS-91. In the qRT-PCR analysis, the expression of this gene gradually increased from 0 to 4 DDT in both genotypes, but the strongest expression was detected in DLS-42—similar to the transcriptome data (Figure 7B and Table 2). Two duplicates of *BrNAC90* (*BrNAC090-1* and *BrNAC090-2*) displayed high stage-specific expression in DLS-91: *BrNAC090-1* at 4 DDT and *BrNAC90-2* at 1 DDT. In addition, *BrNAC090-1* was 10 times more highly expressed in DLS-42 than in DLS-91 at 0 DDT, an observation consistent with the qRT-PCR results (Figure 7B and Table 2). Moreover, *BrNAC092-1* was more highly expressed at 0 and 1 DDT in DLS-42 and at 4 DDT in DLS-91, which was similar to the qRT-PCR data (Figure 7B and Table 2). The varied expressions of *BrNAC* genes at 4 DDT in DLS-91 suggest that these genes are involved in DLS regulation in a stage-specific manner.

In regard to *WRKY* genes, *BrWRKY6-1* FPKM-based read counts and qRT-PCR expression levels were high at 1 and 4 DDT in DLS-91. In contrast, *BrWRKY45* displayed high transcript abundance and qRT-PCR expression at 0 and 1 DDT in DSL-42 and at 4 DDT in DLS-91. Finally, *BrWRKY70-1* was more strongly expressed in DLS-91 than in DLS-42 at all stages according to both transcriptome and qRT-PCR data (Figure 7C and Table 2). We therefore speculate that WRKYs regulate leaf senescence in both genotype- and stage-specific manners.

### 2.8. Genomic Analysis of MADS, NAC, and WRKY TFs

**Figure 8:**
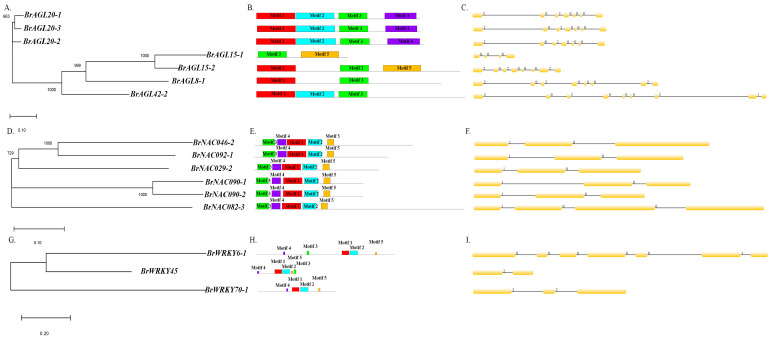
Structural comparison of differentially expressed MADS, NAC, and WRKY transcription factors. (**A**,**D**,**G**) Unrooted neighbor-joining phylogenetic trees of AGAMOUS-LIKE, NAC, and WRKY transcription factors constructed using default parameters in MEGA X. As indicated by scale bars below each tree, branch lengths are proportional to the number of amino acid substitutions per 100 residues, thus reflecting the level of divergence among sequences. Numbers at nodes are bootstrap support values. (**B**,**E**,**H**) Distribution of introns and exons based on the GSDS web tool. The line below each set of introns and exons reflects the gene length. (**C**,**F**,**I**) Conserved motifs predicted using the MEME web tool.

We analyzed evolutionary relationships, intron–exon structures, and conserved motifs of *AGL*, *NAC*, and *WRKY* genes as specified in Materials and Methods (Figure 8). In the phylogenetic tree of *AGL* genes, two sister relationships were uncovered: one between *BrAGL20* genes and one between *BrAGL15* genes (Figure 8A). In terms of *AGL* intron–exon organization, the large intron region and intron phases 0 and 2 were found to be conserved. *BrAGL8-1* and *BrAGL42-2*, which were distinct in the phylogenetic tree, have the highest number of introns (Figure 8B). Five conserved motifs were identified: a SRF-TF motif, two K-box motifs, and two unknown or low-complexity motifs (Figure 8C). Two sister relationships were apparent in the *NAC* gene phylogenetic tree: one involving *BrNAC090* genes, and the other between *BrNAC046-2* and *BrNACL092-1* (Figure 8D). All *NAC* genes except for *BrNAC082-3* were found to have the same number of introns and similar intron phases (0 and 1); *BrNAC082-3*, which did not group with any other *NAC* genes in the phylogenetic tree, possesses three introns (Figure 8E). The six NAC proteins contain four NAM motifs and one unknown or low complexity motif (Figure 8F). One pair of *WRKY* genes, *BrWRKY6-1* and *BrWRKY45*, were sister to one another in the *WRKY* phylogenetic tree (Figure 8G). The three analyzed *WRKY* genes have different intron–exon distributions, different numbers of introns, and different intron phase patterns (Figure 8I). The three predicted WRKY proteins contain two WRKY motifs and three unknown or low-complexity motifs (Figure 8H).

### 2.9. Prediction of MADS, NAC, and WRKY Binding Sites in DEGs and Identification of Other Cis-Elements in Promoter Regions of Selected TFs

**Figure 9:**
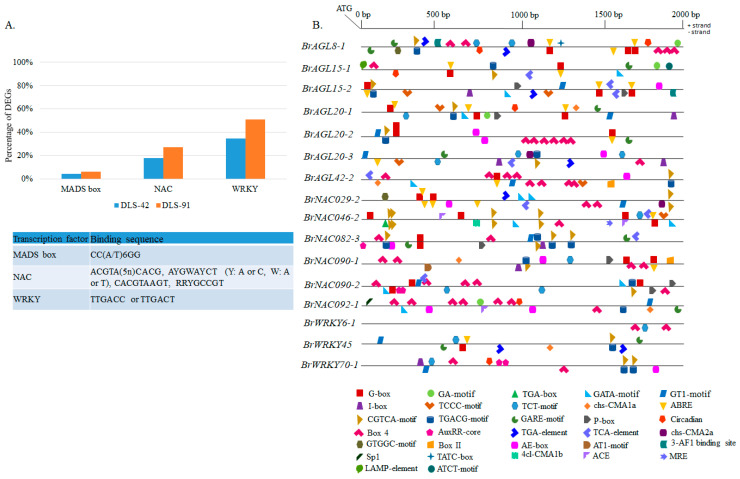
Prediction of *cis*-elements in the 2-kb upstream promoter region of differentially expressed transcription factor genes. (**A**) Proportion of differentially expressed genes from the entire transcriptome data set containing MADS, NAC, and WRKY conserved motifs. The table details the binding sequence of each *cis*-element. (**B**) Schematic representation of conserved motifs identified among AGAMOUS-LIKE, NAC, and WRKY differentially expressed transcription factors. Detailed information on depicted light-related, hormone-related, and other conserved motifs is provided in Tables S12 and S13.

To identify the gene targets of MADS, NAC, and WRKY TFs, we analyzed the 2-kb upstream promoter region in all DEGs (Figure 9A). As a result, we identified 361 genes containing a putative *MADS-box* TF-*binding site* (CArG-box). Some of these genes, such as *BrNAC092-1*, *SAG15*, and *SFP1*, have senescence-related functions (Table S12) [80,81,82]. We also identified putative target genes that interact with NAC TFs via NAM motifs, namely, the photosystem-related genes *CRY2, Psb27-H1, LHCB1.5, PSI-H, LHCB3,* and *LHCB5* (Table S12) [1]. *BrAGL8-1* and *BrAGL42-1*, which were differentially expressed in this study, have conserved NAC motifs as well (Table S12 and Figure 7). The most important of the many genes identified with conserved WRKY motifs were *AGL42*, *NAC029, NAC092, NAC092, FAR1, PHOT2,* and *CRY2*, which have senescence-, light-, and circadian clock-related functions in plants (Table S12) [10,56,65,80,83,84]. A few orthologs of these genes, namely, *BrAGL42-1, BrNAC029-2*, and *BrNAC092-1*, exhibited differential expression in this study (Figure 7). Our results suggest that *BrAGL8-1* and *BrAGL42-1* genes can interact with NAC TFs, and *BrAGL42-1, BrNAC029-1, BrNAC092-1* and *BrNAC092-2* can interact with WRKY TFs, to regulate the expressions of these genes during senescence and other plant growth and development processes.

We also searched for other important *cis*-elements with various functions in the promoter regions of 16 selected MADS, NAC, and WRKY DEGs (Figure 9B). We identified various combinations of *cis*-regulatory elements (CREs) and/or gene-specific CREs. The functionally most important of these CREs were related to light, abscisic acid, methyl jasmonates, gibberellin, auxin, salicylic acid, circadian clock, and other CREs (Table S13). Light-responsive elements (LREs) were also commonly conserved (Table S13). The highest number of LREs in the seven *AGL* genes was found in *BrAGL8-1* and *BrAGL42-2*, most of which were Box 4 (ATTAAT) or G-box ([C/T] ACGT[T/G]) elements. Three *AGL* genes (*BrAGL8-1*, *BrAGL15-1*, and *BrAGL20-1*) contain a circadian motif (CAAAGATATC), with *BrAGL15-1* additionally possessing some gene-specific motifs, such as the ATCT motif (AATCTAATCC) and the LAMP element (CCTTATCCA) (Table S13). In contrast, all NAC TF genes except for *BrNAC090-1* and *BrNAC092-1* have an equal number of light- and hormone-responsive elements. Interestingly, we observed some motifs (GTGGC motif and chs-CMA2a) that are conserved between *BrAGL8-1* and *BrNAC029-2.* A few other LREs and circadian clock-related CREs are also conserved between NAC and AGL family TFs (Table S13). Finally, we found that the three WRKY TFs have different combinations of functional CREs: *BrWRKY6-1* possesses only LREs, whereas *BrWRKY45* has more hormone-related elements than LREs, and *BrWRKY70-1* has an equal number. Circadian clock-related CREs are also conserved in *BrWRKY70-1*. Information on other related conserved CREs is provided in Tables S12 and S13.

## 3. Discussion

Various anabolic and catabolic processes take place during dark-induced leaf senescence. In this study, we compared two genotypes, the control DLS-42 and the rapid-senescence line DLS-91, and identified the physiological and molecular changes causing DLS-91 to more rapidly senesce during dark treatment. We determined the genetic factors—DEGs and TFs—through comparative transcriptome profiling and identified physiological characters by analyzing chlorophyll and photochemical contents. In regard to all these factors, DLS-91 exhibits a broader spectrum of variation compared with DLS-42. Our analysis of *cis*-elements also revealed that *AGL* genes contain both NAC- and WRKY-binding sites. Although many previous studies have focused on the role of *NAC* and *WRKY* TF genes, our results more strongly suggest the involvement of *AGL* genes in the rapid senescence of DLS-91. We thus speculate that *AGL* genes, along with *NACs* or *WRKYs*, regulate dark-induced rapid senescence in *B. rapa* DLS-91. The physiological and molecular parameters analyzed here are likely to be critical functional components involved in the rapid leaf senescence of DLS-91 [46,47,49,54,56,65,85].

### 3.1. Physiological and Molecular Responses of DLS-91 during DLS

Leaf yellowing, a distinct initial symptom of leaf senescence, is caused by chlorophyll depletion [51]. This reduction of chlorophyll is a characteristic reaction in the breakdown of photoreceptors during light depletion [1,86]. In the present study, we compared the chlorophyll content and photochemical efficiency (Fv/Fm) of leaf samples of DLS-42 and DLS-91 at three different phases of dark treatment (Figure 2A,B). The rapid loss of chlorophyll and reduction in photochemical efficiency observed in DLS-91 may have been due to the breakdown of photosynthetic components leading to diminished photosynthesis during dark treatment [1,86]. Similarly, leaves of Arabidopsis and barley subjected to dark treatment have been reported to exhibit a decline in chlorophyll and the Fv/Fm ratio according to the progression of dark exposure [11,85]. Genes related to chlorophyll biosynthesis (*PORC, PORB, GSA1, HEMF1,* and *HEMB1*) and degradation (*CLH1*) were also highly expressed in DLS-91 before dark treatment (i.e., 0 DDT) and then displayed reduced expression, which indicates that the high rate of chlorophyll reduction resulted in the rapid senescence of DLS-91 (Figure 1, Figure 2A and Figure 5A). We then used the leaf-senescence markers *EIN2*, *ORE1*, and *SAG12* to analyze DLS-91-specific expression patterns during dark treatment [1,32,50]. According to the expression analysis of orthologous genes, the expression of *BrSAG12-1* at 2 DDT was higher in DLS-91 compared with DLS-42. These data were correlated with physiological parameters, thus indicating that *BrSAG12-1* is a genotype-specific early leaf senescence regulator in *B. rapa* (Figure 2C and Figure S3). Because *SAG12* is controlled by developmental signals and not induced by various stress conditions in Arabidopsis*,* this gene is believed to be a reliable marker for natural leaf senescence [87,88]. As shown in Figure 2C, however, the *BrSAG12* gene is strongly induced during dark treatment in *B. rapa.* Previous studies have also reported that high accumulation of SAG12 in early-senescent leaves is due to dark treatment [1,89]. Given the phenotypic, physiological, and molecular evidence, we therefore hypothesize that leaf senescence can vary according to genotype in *B. rapa*.

### 3.2. Roles of MADS, NAC, and WRKY TFs in Various Processes Related to Senescence

In general, TFs act as central regulators of various processes, including leaf senescence [4,89,90], but no transcriptomic studies focused on TFs related to leaf senescence in *B. rapa* have been reported. In the present study, we identified 848 genes, including 15.6% encoding TFs, that were differentially expressed in DLS-91 at different stages of DLS (Figure 3; Tables S4 and S5). Many TFs, including NAC (21.8%), WRKY (18%), and MADS (9.8%) TFs (Table S5), were differentially expressed, which indicates their significant roles in various physiological and biochemical processes during leaf senescence [89,90,91] and confirms the necessity of studying TFs regulating leaf senescence in *B. rapa*.

Our transcriptome analysis uncovered 10 *AGL*-*MADS-box* genes (*BrAGL8, BrAGL15, BrAGL20,* and *BrAGL42*) that are orthologs to Arabidopsis genes *AtAGL8* [46]*, AtAGL15* [47]*, AtAGL20* [64], and *AtAGL42* [65] that function in senescence according to mutant and transgene expression analyses. Among the three copies of *BrAGL8*, *BrAGL8-1* had the highest transcript abundance (FPKM values) at all three sampled stages in DLS-91 compared with DLS-42. We further validated the expression of this gene by qRT-PCR and obtained results similar to those of the transcriptome analysis (Figure 6 and Table S10). In Arabidopsis, the flowering time regulator *AGL-8* is also expressed in rosette and cauline leaves [52,92,93]. Interestingly, the promoter regions of *BrAGL8-1* and *BrNAP/BrNAC029-2* both possess a common LRE (the GTGGC motif), and we also discovered putative NAC (AGATTCGT) and MADS-box (CCTTTTTTGG) binding sequences in the promoter regions of both genes (Tables S12 and S13). Previous investigations on *SEPALLATA3* have revealed that this gene along with AGL-9 TF binds to the *ANAC092* promoter, which suggests that NAC genes can control AGL-MADS TF regulation of various functions [29,94]. In the case of *BrAGL15*, *BrAGL15-1* was only expressed at all stages in DLS-91 according to qRT-PCR and transcriptome data. In contrast, the expression of *BrAGL15-2* did not differ between DLS-42 and DLS-91. The variation in the expressions of these two gene duplicates may be due to structural differences, as indicated by their phylogenetic positions as well as the arrangement of their motifs, introns, and exons (Figure 6 and Figure 7A). Previous studies of Arabidopsis have shown that *AGL15* increases the longevity of floral organs by delaying their senescence and that overexpression of this gene does not delay leaf senescence [47,95]. Consequently, *BrAGL15-1* and *BrAGL15-2* may have genotype- and non-genotype-specific functions during dark-induced senescence. The three paralogs of *BrAGL-20/SOC1* (*BrAGL20-1*, *BrAGL20-2*, and *BrAGL20-3*) exhibited a wide range of expression patterns (Figure 6 and Figure 7A). Among them, *BrAGL20-2* was highly expressed in DLS-42 at all stages. Sequence analysis uncovered differences in the structure, phylogenetic placement, and first- and second-intron sizes of *BrAGL20-2* compared with the other two paralogs. Interestingly, the *BrAGL20-2* gene promoter region contains a putative WRKY binding sequence (Table S12). Arabidopsis *SOC1* mutants display inhibited Chl degradation and retain maximum photochemical efficiency, which suggests that SOC1 negatively regulates the general senescence process [65]. *BrAGL42* gene duplicates were highly specifically expressed in DLS-42 according to our qRT-PCR and transcriptome analyses. Interestingly, the *BrAGL42-1* gene includes both NAC (ACGTATCT) and WRKY(AGTCAA) binding sequences in its promoter region. Arabidopsis *AGL42/FYF* mutants exhibit delayed floral senescence/abscission due to repression of ethylene responses [65]. Taken together, these results suggest that the *B. rapa AGL* orthologs *BrAGL-20-2*, *BrAGL-42-1*, and *BrAGL-42-2* play crucial, genotype-dependent roles during DLS.

The NAC TF genes *AtNAP/ANAC029* [96], *ANAC046* [31], *VNI1/ANAC082* [66], and *ORE1/ANAC092* [80] have various functions related to leaf senescence in Arabidopsis. In the present study, all of the orthologs of these genes, except for *BrNAC082-3,* were more highly expressed in DLS-91 than in DLS-42 at late stages of dark treatment (4 DDT). In addition, *BrNAC029/NAP* and *BrNAC092/ORE1* were highly expressed before (0 DDT) and at the early stage (1 DDT) of dark treatment in DLS-91, whereas *BrNAC082-3* was more highly expressed in DLS-42 than in DLS-91 at all dark-treatment stages. According to our analysis of *BrNAC082* sequences, *BrNAC082-3* is phylogenetically distinct and has more introns than other *NAC* genes (Figure 6 and Figure 7B). Similarly, *BrNAC090-1* and *BrNAC090-2* were highly expressed at 0 DDT in DLS-42 and at 1 DDT in DLS-91. The variation in the expressions of these two genes may be due to structural differences (Figure 6 and Figure 7B). An earlier study of Arabidopsis mutants of *ANAC017, ANAC08*2, and *ANAC090* demonstrated that these “NAC troika” genes negatively regulate leaf senescence by controlling salicylic acid and reactive oxygen species responses [66]. As mentioned above, conserved binding sites of MADS-box and WRKY TFs are present in the promoters of these NAC genes. Furthermore, most of these genes are highly expressed in DLS-91 leaves at the late stage (4 DDT) of senescence (Table S10). These results indicate that NAC genes have a regulatory role in DLS at the late stage of senescence in DLS-91.

Among the three validated *BrWRKY6* orthologs, the expression of *BrWRKY6-1* was slightly elevated at 1 and 4 DDT in leaves of DLS-91 than DLS-42. A study involving an Arabidopsis mutant has shown that *WRKY6* positively regulates senescence through interaction with the senescence-induced receptor-like kinase (SIRK) gene [76]. *BrWRKY6* may function during dark-induced senescence in *B. rapa*, but further functional analysis is needed to support our finding of stage/genotype-specific expression. In contrast, the expression of *BrWRKY45* varied only slightly between the two genotypes. As a critical component of the GA-mediated signaling pathway, WRKY45 functions to positively regulate age-triggered leaf senescence [35]. In regard to *BrWRKY70*, *BrWRKY70-1* was highly expressed in DLS-91 at all sampled stages. Similar to *BrWRKY70-1*, *BrWRKY70-2* was strongly expressed at all stages, whereas *BrWRKY70-3*, whose promoter contains two NAC-binding sequences (ATGAATCT and AGGTACAT; Table S12), was only highly expressed at 1 DDT in DLS-91. Among the three paralogous WRKY54 genes, *BrWRKY54-2* was highly expressed in DLS-91. Previous studies in Arabidopsis have shown that *WRKY54* and *WRKY70* work together to negatively regulate leaf senescence [78]. Finally, other *WRKY* genes were more strongly expressed in DLS-91 during dark treatment (1 and 4 DDT) (Table S10). Although these results suggest that these genes also have major roles during DLS in DLS-91, additional functional studies are needed to support the expression data (Figure 6 and Table S10).

## 4. Materials and Methods

### 4.1. Estimation of Chlorophyll Content and Selection of Plant Materials

A core collection of 177 *B. rapa* lines was cultivated in a growth chamber at 23 °C under a 16 h light photoperiod. The *B. rapa* seedlings were grown in soil for 4 weeks, and their fourth leaves were then detached for the dark treatment. The detached leaves were incubated for 1 to 9 days in Petri dishes wrapped in aluminum foil and containing a floral foam block soaked in distilled water (Figure S1). For chlorophyll extraction, the detached leaves were subsequently incubated in 95% ethanol for 17 h in darkness. The extracts were then centrifuged at 12,000× *g* for 5 min at 4 °C. Chlorophyll was quantified by measuring absorbance at 645 and 663 nm, and chlorophyll content was calculated as the ratio of ([20.23 × A_645_] + [8.023 × A_657_]) g^−1^ fresh weight [1,51]. On the basis of their leaf chlorophyll content at 3 and 7 DDT (as explained in results) the control line DLS-042 and the rapid-senescence line DLS-091 were selected for further analysis (Figure S2). At least four biological replicates were used for the chlorophyll estimation.

### 4.2. Photochemical Efficiency

To estimate DLS-42 and DLS-91 photochemical efficiencies, healthy, fully grown fourth leaves were detached from 4-week-old plants and subjected to dark treatment. A Handy PEA meter (Hansatech, Norfolk, UK) was used to measure Fv/Fm values of the dark-treated leaves according to the manufacturer’s instructions. At least five biological replicates were used for the estimation of Fv/Fm values.

### 4.3. RNA Sequencing and Transcript Annotation

DLS-42 and DLS-91 plants were grown for 1 month, with fourth leaves then detached from three biological replicates and subjected to dark treatment. After 0, 1, and 4 DDT, total RNA was extracted from leaves of 18 samples (2 lines × 3 stages × 3 biological replicates) using an RNeasy Mini kit (Qiagen, Seoul, Korea) and sequenced on a Illumina Hi-seq2000 platform by Seeders, Inc. (Daejeon, Korea). The sequence data have been submitted to the NCBI database and are available under the accession number PRJNA723680. The resulting RNA sequences were analyzed according to Rameneni et al. [97] After raw reads were trimmed using the Dynamic-Trim and Length-Sort tools in the Solexa QA package [98], clean reads were assembled with Velvet v1.2.08 and Oases v0.2.08 [99]. The assembled transcripts were functionally annotated using BRAD [100] and KEGG databases. Bowtie2 v2.1.0 software was used for mapping of transcripts [101]. To calculate gene expression values, transcript normalization was performed with the R package DESeq [102].

### 4.4. Identification of DEGs and TFs and GO Annotation

DEGs were selected according to their log_2_ FC values. In this study, genes with log_2_ FC > 1 or log_2_ FC < −1 were considered to be upregulated or downregulated, respectively [97]. For identification of TFs, we downloaded all 4127 *B. rapa* TFs in the Plant Transcription Factor Database (http://planttfdb.cbi.pku.edu.cn/, accessed on 17 March 2021) [103]. All of the assembled transcripts were compared against the downloaded TF data using BlastX software.

GO alignment [104] of the complete set of transcripts was carried out using the agriGO database. Graphs were generated based on default parameters, and the transcripts were classified into three functional categories: biological process, cellular component, and molecular function. To predict the functional characteristics of upregulated and downregulated genes, *p*-values computed from Fisher’s exact test were used to identify significant associations between genes and GO terms, and a KEGG enrichment analysis was performed using the KOBAS online tool (http://kobas.cbi.pku.edu.cn/kobas3, accessed on 17 March 2021) [105] to obtain pathway annotations for each transcript.

### 4.5. Validation of Transcript Abundance by qRT-PCR

To validate gene expression levels estimated from the transcriptome data, we selected 16 functionally characterized genes exhibiting variation in expression. Total RNA was isolated using an RNeasy mini kit (Qiagen) from 0-, 1-, and 4-DDT leaves of DLS-42 and DLS-91. A DNAse kit (Qiagen) was used to digest genomic DNA. cDNA was synthesized using a ReverTra Ace-α kit (Toyobo, Japan) and used as a template for qRT-PCR analyses on a CFX96 Real-Time system (Bio-Rad, Hercules, CA, USA). The qRT-PCR analysis was conducted on 13 SAGs with three biological replicates and three technical replicates using the following cycling conditions: 95 °C for 3 min, followed by 39 cycles of 95 °C for 15 s and 58 °C for 20 s [97]. Relative expression levels were determined by normalizing the data according to the comparative 2^−ΔΔCt^ method [106] using actin and EF-1α genes as a reference.

### 4.6. Promoter Analysis

The 2-kb region upstream of the transcription start site of selected DEGs was extracted, and all conserved *cis*-regulatory motifs were predicted using the plantCARE database (http://bioinformatics.psb.ugent.be/webtools/plantcare/html/, accessed on 17 March 2021) [107].

### 4.7. Genomic Analysis

A multiple alignment of the 16 protein sequences was generated in Clustal X [108] and used to generate an unrooted tree by the distance-based neighbor-joining method in MEGA X [109] with 1000 bootstrap iterations. Conserved motifs were identified using the MEME suite v5.3.3 (https://meme-suite.org/meme/, accessed on 17 March 2021) [110]. Analysis of gene intron–exon structural variation based on genomic and coding sequences was conducted using the GSDS v2.0 online tool (http://gsds.gao-lab.org/Gsds_about.php, accessed on 17 March 2021) [111].

## 5. Conclusions

Here, we showed for the first time, to our knowledge, the possible DLS underlying mechanism (s) in *B. rapa*. According to phenotypic, chlorophyll content, and photochemical efficiency data, DLS-91 undergoes more rapid leaf senescence than DLS-42 during dark treatment. Analysis of transcriptome- and qRT-PCR-based expression data demonstrated that the DLS-91-specific expression of most *AGL*-type MADS TFs varies at different stages of leaf senescence. In addition, NAC and WRKY binding sequences are conserved in the promoter regions of *AGL* genes. The discovery of an expression pattern specific to the rapid-DLS line DLS-91 provides essential information for identifying genes involved in dark-induced leaf senescence in *B. rapa*. Further functional analyses of these candidate genes are required to reveal the molecular mechanism underlying DLS.

## Figures and Tables

**Table 1:** Genes identified from the transcriptome data and involved in various functions during leaf senescence.

Functional Category	Gene Count	Expression Value (Log2 Fold Change Range)			Reference
		DLS-91_0d_vs_ DLS-42_0d	DLS-91_1d_vs_ DLS-42_1d	DLS-91_4d_vs_ DLS-42_4d	
Chlorophyll biosynthesis genes	44	1.94~−1.28	1.30~−3.79	2~−2.96	[5]
Chlorophyll degradation genes	11	6.07~−1.88	1.22~−1.35	1.02~−1.45	[5]
Subunits of photosystems I genes	13	2.67~−0.81	1.85~−1.76	2.16~−1.71	[5]
Subunits of photosystems II genes	42	0.73~−5.40	1.35~−2.50	2.25~−2.85	[5]
Regulators that act through changes in chromatin	11	0.58~−0.42	0.63~−0.71	0.76~−0.52	[57,59]
Regulators that act on the transcriptional level	45	2.78~−5.12	2.85~−2.23	3.90~−3.92	[57,59]
Regulators that act at the translational level	1	1.54	−0.90	−0.65	[59]
Regulators that act at the post-translational level	25	2.61~−5.35	2.60~−4.74	2.29~−6.03	[57,59]
Phytohormone signal components in leaf senescence and plant immunity	29	1.66~−5.72	2.09~−5.53	1.59~−5.95	[55]
List of the genes that are involved in leaf senescence and plant immunity.	33	2.18~−3.83	2.94~−3.31	2.51~−2.93	[58]
TFs Associated with Leaf Senescence in Crops	27	0.5~−2.58	1.33~−2.67	2.03~−1.15	[3]
List of genes that delay leaf senescence	119	4.59~−1.72	4.64~−3.78	5.63~−5.44	[3,58]
List of gene involved in leaf color change	74	4.47~−6.84	4.55~−5.99	3.48~−6.22	[58]
Previously reported senescence up regulated genes during dark-induced and age-triggered senescence	1218	7.16~−7.42	7.90~−8.03	8.27~−6.83	[5]
Previously reported senescence down regulated genes during dark-induced and age-triggered senescence	1310	8.09~−6.31	7.52~−5.75	8.18~−5.87	[5]

**Table 2:** Annotations of genes selected for qRT-PCR validation and their senescence-related functions.

Given Name	Gene Id V 3.0	DLS-42_0d	DLS-42_1d	DLS-42_4d	DLS-91_0d	DLS-91_1d	DLS-91_4d	Chromosome	Gene Start	Gene End	Effect on Senescence	Source	Reference
*BrAGL8-1*	BraA02g042750	1.63	2.06	2.46	26.29	14.48	27.51	A02	25192486	25195876	Promote	Mutant	[46]
*BrAGL15-1*	BraA09g025840	0.00	0.00	0.00	9.40	8.36	16.52	A09	17735929	17736685	Delay	Transgene	[47]
*BrAGL15-2*	BraA10g025090	0.16	0.98	1.33	0.51	0.39	0.61	A10	12699925	12701540	Delay	Transgene	[47]
*BrAGL20-1*	BraA03g023790	0.98	1.19	1.23	0.00	3.97	2.48	A03	10918286	10920672	Promote/Delay	Mutant	[65]
*BrAGL20-2*	BraA04g031640	85.00	29.39	30.50	3.71	1.66	2.40	A04	18723546	18725960	Promote/Delay	Mutant	[65]
*BrAGL20-3*	BraA05g005370	14.22	13.90	16.29	3.07	7.51	11.53	A05	2530305	2532747	Promote/Delay	Mutant	[65]
*BrAGL42-2*	BraA09g007170	32.44	18.26	13.67	0.17	0.12	0.04	A09	3574619	3579987	Delay	Transgene	[47]
*BrNAC029-2*	BraA07g034350	95.51	170.36	235.98	10.82	34.48	377.56	A07	21638992	21639971	Promote	Mutant/Transgene	[10,69]
*BrNAC046-2*	BraA05g042320	1.66	1.41	4.08	0.68	2.27	7.89	A05	24529701	24531081	Promote	Mutant/Transgene	[33]
*BrNAC082-3*	BraA10g031140	56.95	60.77	71.15	32.97	32.16	49.86	A10	14081664	14083371	Delay	Mutant/Transgene	[10,71]
*BrNAC090-1*	BraA03g010320	12.98	2.45	0.09	0.77	2.05	1.09	A03	4319782	4321055	Delay	Mutant/Transgene	[67]
*BrNAC090-2*	BraA10g018990	1.05	0.19	0.09	0.57	2.68	0.24	A10	9862356	9863361	Delay	Mutant/Transgene	[67]
*BrNAC092-1*	BraA04g012020	16.54	33.84	31.40	0.35	9.03	61.35	A04	7538248	7539477	Promote	Mutant	[30,31]
*BrWRKY6-1*	BraA03g063940	0.16	0.65	0.16	0.02	1.64	0.41	Scaffold000096	241928	244473	Promote	Mutant	[77]
*BrWRKY45*	BraAnng005520	9.18	76.23	70.30	3.02	56.72	95.61	Scaffold000217	68236	68758	Promote	Mutant	[78]
*BrWRKY70-1*	BraA04g004020	8.24	48.80	1.22	47.34	343.66	20.64	A04	2296461	2297781	Delay	Mutant	[80]

## Supplementary Materials

The following are available online at https://www.mdpi.com/article/10.3390/ijms22116017/s1.

## Abbreviations

DDT	Days after dark treatment
DEGs	Differentially expressed genes
TFs	transcription factors
SAGs	Senescence associated genes
FPKM	Fragments per kilo base of transcript per million fragments mapped
FC	Fold change
MA plot	Bland–Altman plot
GO	Gene ontology
