# Supplementary figures and images for: Comparative Transcriptome-Based Mining of Senescence-Related MADS, NAC, and WRKY Transcription Factors in the Rapid-Senescence Line DLS-91 of Brassica rapa
Source: Int J Mol Sci. 2021 Jun 2;22(11):6017. doi: 10.3390/ijms22116017 (PMC8199657; doi:10.3390/ijms22116017)

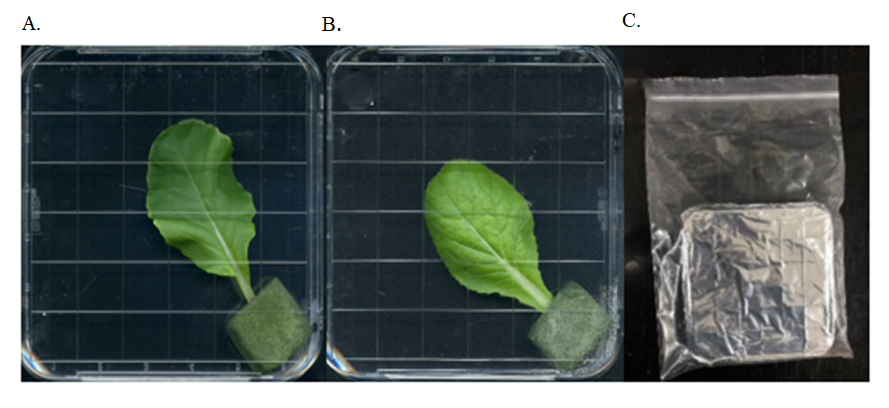

Supplement: Supplementary file 1 [file ijms-22-06017-s001.zip › data/Fig. S1.tif]

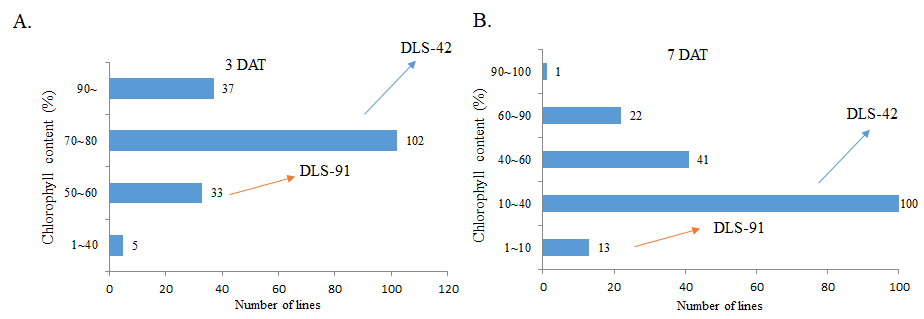

Supplement: Supplementary file 1 [file ijms-22-06017-s001.zip › data/Fig. S2.tif]

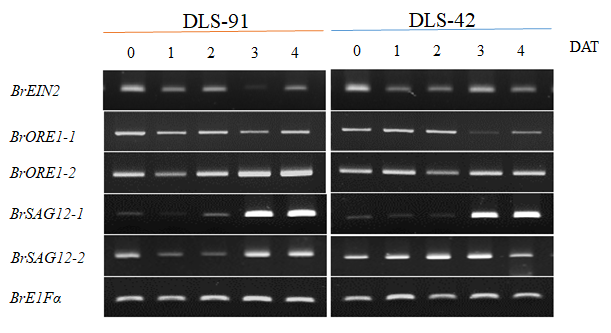

Supplement: Supplementary file 1 [file ijms-22-06017-s001.zip › data/Fig. S3.tif]
